# An integrated mediapipe-optimized GRU model for Indian sign language recognition

**DOI:** 10.1038/s41598-022-15998-7

**Published:** 2022-07-13

**Authors:** Barathi Subramanian, Bekhzod Olimov, Shraddha M. Naik, Sangchul Kim, Kil-Houm Park, Jeonghong Kim

**Affiliations:** 1 School of Computer Science and Engineering, Kyungpook National University, Buk-gu, Daegu, 41566 South Korea; 2 Division of Computer Engineering, Hankuk University of Foreign Studies, Seoul, South Korea; 3 School of Electronics Engineering, Kyungpook National University, Buk-gu, Daegu, 41566 South Korea

**Keywords:** Computer science, Information technology

## Abstract

Sign language recognition is challenged by problems, such as accurate tracking of hand gestures, occlusion of hands, and high computational cost. Recently, it has benefited from advancements in deep learning techniques. However, these larger complex approaches cannot manage long-term sequential data and they are characterized by poor information processing and learning efficiency in capturing useful information. To overcome these challenges, we propose an integrated MediaPipe-optimized gated recurrent unit (MOPGRU) model for Indian sign language recognition. Specifically, we improved the update gate of the standard GRU cell by multiplying it by the reset gate to discard the redundant information from the past in one screening. By obtaining feedback from the resultant of the reset gate, additional attention is shown to the present input. Additionally, we replace the hyperbolic tangent activation in standard GRUs with exponential linear unit activation and SoftMax with Softsign activation in the output layer of the GRU cell. Thus, our proposed MOPGRU model achieved better prediction accuracy, high learning efficiency, information processing capability, and faster convergence than other sequential models.

## Introduction

Sign language is a vision-based interactive language with unique and complex linguistic rules. It is used by people who are hearing impaired to communicate and exchange their feelings, ideas, and thoughts using various parts of the body^1,2^. Since sign language has a unique linguistic structure, it differs from one place to another according to its geographic location^3^. Each country has developed its sign language for communication among its deaf and hard-of-hearing communities^4^. Some of the popular sign languages are American sign language (ASL) in the US^5,6^, British sign language in the UK, Indian sign language (ISL) in India^7,8^, Korean sign language in Korea^9^. From the World Health Organization report, approximately 500 million people worldwide suffer from hearing loss^10,11^. Because of the high prevalence of the hard-of-hearing community population, there has been an increased interest in eliminating communication obstacles faced within the hard-of-hearing community and other people with normal hearing^12^.

Sign language recognition (SLR) develops an assistive system that automatically converts an input sign into its corresponding speech or text^13^. Thus, the SLR system is useful for overcoming the communication gap between hearing and nonhearing communities and creates a new path for human-computer interaction-based applications^14–18^. The major challenge to developing a continuous SLR system is finding a modeling prototype that acquires the sign gesture and its corresponding text. Starner et al.^19^ developed a video-based real-time continuous SLR system using a single camera with 40 vocabulary signs of ASL sentences using a hidden Markov model (HMM) classifier. Similarly, Vogler and Metaxas^20^ developed a continuous SLR system using three orthogonally positioned cameras to mitigate the problems caused by occlusion and uncontrolled movements in ASL sentences. HMM was used for the recognition process with a vocabulary of 53 signs, and the system was tested on 97 sign sentences, producing a recognition rate of 92.11$\%$ and 95.83$\%$, respectively. From the above literature survey, it is crystal clear that all sensor and vision-based techniques are more restrictive and cost effective^21,22^. Although different techniques are available, the challenges of hand tracking^23,24^, occlusion of hand movements^25^, high computational cost^26^, feature selection^27^ and lower learning efficiency^28^ still exist.

To address these drawbacks, we proposed a MOPGRU SLR system that diminishes the problem of hand occlusion and lower learning efficiency by adjusting the output of the update gate using the reset gate integrated with an open-source framework called the MediaPipe Holistic pipeline^29^ . Furthermore, we changed the activation function in the output layer and candidate memory state of each GRU cell to achieve a faster, simpler, and cost effective SLR system. The main contributions of this study are summarized as follows:

- We proposed a novel MOPGRU model that calibrate the resultant of the update gate by the reset gate, which enchances the learning process of the GRU gating unit, thereby accelerating the convergence rate, eliminating gradient depletion problem, and improving the learning efficiency.
- We replaced the hyperbolic tangent (Tanh) activation function in the candidate memory state with an ELU activation function to overcome the vanishing and exploding gradient problem, and further enhance the model. As a result, we obtained lower training time and good performance for the MOPGRU model than those of other variants. Additionally, the activation function of the output layer of each GRU cell was replaced with Softsign instead of SoftMax to reduce the computational complexity and hence, the training time of the model.

The rest of this paper is organized as follows: In “Related work” section introduces existing SLR methods and their limitations. In “Proposed methodology” section introduces and describes the proposed methodology. In “Experimental results and discussion” section presents the experimental settings, results, comparison of our method with other methods, and analyzes the model performance. Finally, “Conclusion” section presents our contributions and outlines the future work.

## Related work

Previous researchers have emphasized their work on the prediction of sign language gestures to support people with hearing impairments using advanced technologies with artificial intelligence algorithms. Although much research has been conducted for SLR, there are still limitations and improvements that need to be addressed to improve the hard-of-hearing community^30^. This section presents a brief literature review of recent studies on SLR using sensor and vision-based deep learning techniques.

### Sensor-based deep learning techniques

To bridge the communication gap between the hard-of-hearing community and normal people, researchers have proposed a real-time ISL hand gesture recognition system that uses a Microsoft kinetic RGB-D camera for inputting images and applies deep learning techniques to achieve one-to-one mapping between the depth and RGB pixels on training over 45,000 RGB and depth images, while achieving a prediction accuracy of 98.81$\%$^31^. Although the model resulted in good accuracy, it emphasized the need for a large dataset with more images to train and a high-pixel RGB camera. Like the aforementioned model, an algorithm with a support vector machine and Microsoft kinetic Xbox 360 RGB images for translating Indian sign language gestures into English text and speech with 100$\%$ prediction accuracy for the signs representing one numeric value and six ISL alphabets alone was proposed^32^.

Using an expensive leap motion controller [LMC]^26,30^, researchers have proposed a training method for ASL with an long short-term memory (LSTM) recurrent neural network for handling a sequence of input and yields an average accuracy rate of 91.08$\%$. This proposed model has several limitations due to the leap motion controller because the number of users affects the model accuracy. Furthermore, this method is limited to recognizing only one hand gesture. Neethu et al.^33^ introduced a deep convolutional neural network (CNN) classification approach with a connected component analysis algorithm to segment the fingertips from the hand image and classify only eight different gestures into various classes with a 96.2$\%$ recognition rate. The performance was analyzed only in terms of sensitivity, accuracy, and recognition rate.

Gupta et al.^28^ proposed a sensor-based multilabel classification to categorize ISL isolated signs by processing signals from sEMG and IMUs placed on both the forearms of signers in an integrated manner with some classification and categorization errors. Similarly, Salem et al.^34^ proposed a real-time customize glove-based method with five-flex and one accelerometer sensor to recognize Arabic sign language gestures and display corresponding English text and audible sounds. Generally, motion gloves sign language prediction has high limitations in terms of hand tracking and is uncomfortable for users compared to vision-based methods. Like leap motion controllers, they are expensive, time-consuming, and may produce inaccurate calibrations due to wear and tear from the frequent usage of gloves.

### Vision-based deep learning techniques

Rastgoo et al.^35^ proposed a real-time isolated hand SLR (IHSLR) from an RGB video^36^ by combining deep learning models, singular value decomposition (SVD), and SSD with LSTM with ResNET50^37^ and further with SSD, 2DCNN, 3DCNN^38^, and LSTM to obtain features from the 3D hand coordinators and achieved a high accuracy of 99$\%$^25^. The proposed model is simple and fast; however, the model is not able to recognize in case of high inter-class similarities, and in some cases there also exists some misclassification because of the high occlusion of two hands in some signs, making it difficult to predict hand signs correctly. Similarly, Chen et al.^39^ proposed a three-tier network architecture with the short-term traffic prediction model built by using LSTM to simplify network management and to reduce communication overheads. Hurroo et al.^24^ proposed a convolutional neural network (CNN) with the HSV color algorithm and various computer vision techniques for recognizing only 10 American Sign gesture alphabets and obtained an accuracy of 90$\%$. Action recognition architectures constructed with 3D CNN models such as I3D^40^ architecture is also used for SLR task in^41^. Although this method uses low computing power, robustness is not achieved, and the prediction accuracy is lower than that of other CNN models.

Ojha et al.^42^ implemented a fingerspelling sign language translator using a CNN to detect ASL and translate its corresponding text and speech in real-time. The proposed model achieved an accuracy of 95$\%$ with certain limitations; for example, when running the project, the threshold must be monitored to avoid distorted grayscale in the frames if it does not lead to resetting the histogram or looking for appropriate lighting conditions. Additionally, several extensive literature on train CNNs for continuous SLR with weakly labeled data has been reported^43^. Here, the CNN inside an iterative expectation-maximization algorithm was trained with over 1 million poorly labeled hand gesture images representing the sign language. However, moderate prediction accuracy was achieved despite using only the prerecorded pictures and videos as the input, which are unsuitable for real-time hand gesture recognition.For continuous SLR with deep learning^44^, a heuristic approach for epenthesis detection to support continuous natural communication between the machine and user was proposed. Although they have reduced classification confusion, they showed good results only when tested individually with more resources needed for implementing an integrated continuous SLR system.Likewise, several studies exist where both static and dynamic gesture recognition were performed using machine learning and deep learning methods^9,45–52^.

### Standard GRU

**Figure 1:**
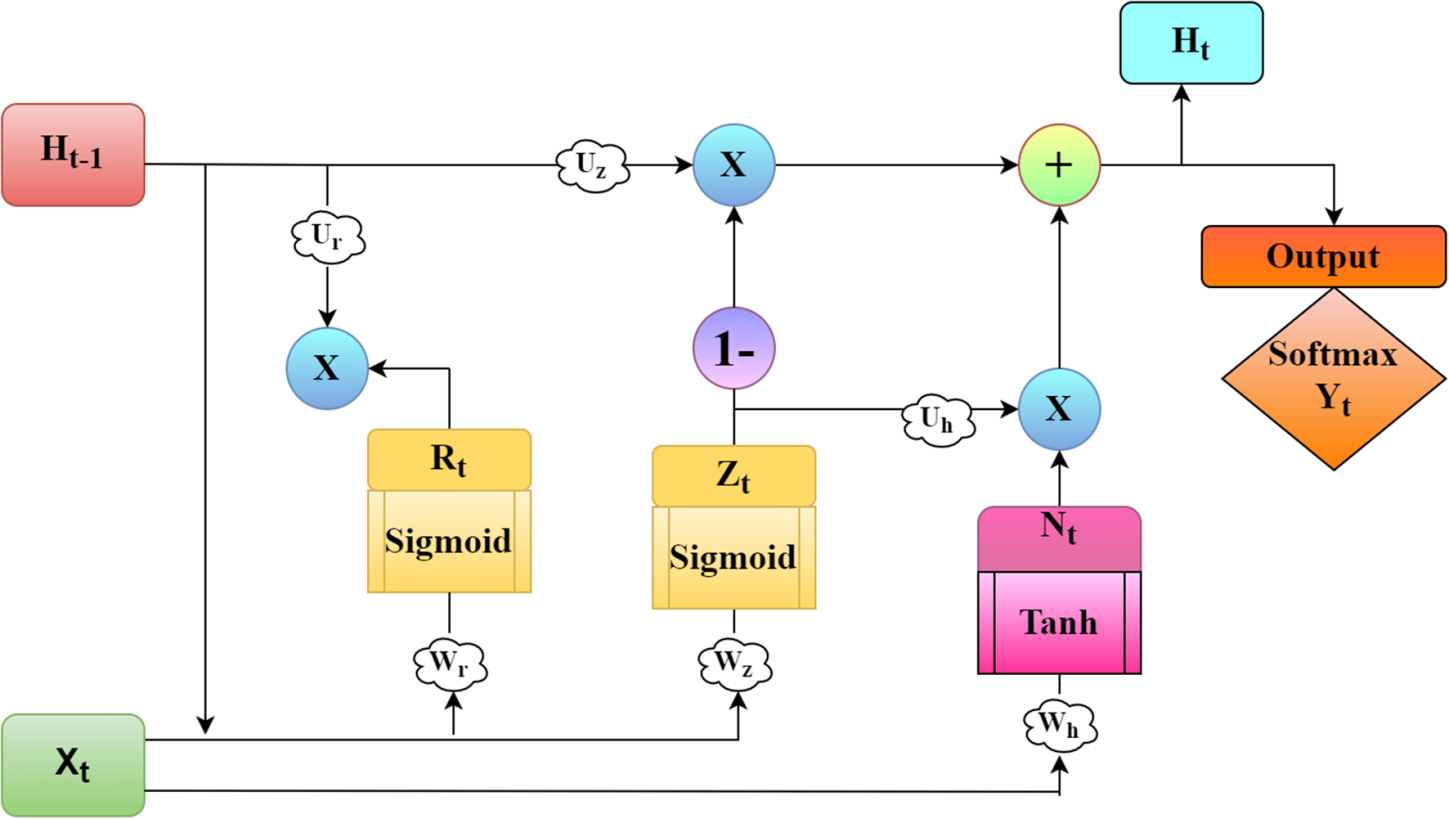
Structure of a standard GRU cell.

Most computer vision problems require handling temporal dependencies among inputs and modeling short-term and long-term sequences. Recurrent neural networks (RNNs) are efficient in managing and processing such sequential data. Compared to traditional neural networks, RNNs focus on manipulating state neurons to learn contextual relations in and between sequential data^53^. Training RNNs is a difficult task due to several limitations and the vanishing and exploding gradient problems. GRUs were applied to solving the vanishing and exploding gradients incorporated into conventional RNNs^54,55^. Among the RNNs, the most frequently used are LSTM networks that have achieved state-of-the-art performance on various deep learning and machine learning tasks. As a variant of LSTM, GRU performs equally as an LSTM and produces good results. It enhances the configuration of the LSTM units and conjugates the three gating units to two gating units of the LSTM as update gate and reset gate. Thus, the parameters of the GRU network model are considerably less, thereby sustaining information dependency and reducing the training time. Figure 1 shows the general structure of a standard GRU cell.

From Fig. 1, at each time step t, a GRU cell takes the contents of previous hidden state $H_{t-1}$ and present input $X_{t}$, operates them through reset and update gates, and passes the computed current state $H_{t}$ to the next time step. The general formulas of a standard GRU cell are as follows:

1 $$\begin{aligned} R_{t}=\, & {} \delta (W_{r}\cdot X_{t}+U_{r}\cdot H_{t-1}+B_{r}) \end{aligned}$$

2 $$\begin{aligned} Z_{t}= & {} \delta (W_{z}\cdot X_{t}+U_{z}\cdot H_{t-1}+B_{z}) \end{aligned}$$

3 $$\begin{aligned} N_{t}= \,& {} \tanh (W_{h}\cdot X_{t}+U_{h}\cdot (H_{t-1}\odot R_{t})+B_{h}) \end{aligned}$$

4 $$\begin{aligned} H_{t}=\, & {} Z_{t} \odot N_{t} + (1-Z_{t}) \odot H_{t-1} \end{aligned}$$

5 $$\begin{aligned} Y_{t}= \,& {} SoftMax((W_{o}*H_{t})+B_{o}) \end{aligned}$$

6 $$\begin{aligned} \delta (x)=\, & {} \frac{1}{1+e^{-x}} \end{aligned}$$

7 $$\begin{aligned} tanh(x)= \,& {} \frac{e^{x}-e^{-x}}{e^{x}+e^{-x}} \end{aligned}$$

where $R_{t}$ and $Z_{t}$ denotes the resultant of the reset and update gates at time *t* in Eqs. (1) and (2), respectively. $N_{t}$ in Eq. (3) denotes a current candidate memory value vector that is computed from a hyperbolic tangent activation function (*tanh*), represented in Eq. (7) at time *t*. $H_{t}$ in Eq. (4) indicates the resultant of the standard GRU unit at time $t-1$, and computed as a linear interpolation of previous states $H_{t-1}$ and $N_{t}$ using the result from $Z_{t}$ in Eq. (2). The Sigmoid activation ($\delta$) in Eq. (6) is applied to both $R_{t}$ and $Z_{t}$ gates to scale the values within 0 and 1. $\odot$ denotes the Hadamard product which is nothing but element-wise multiplication. $X_{t}$ is the current input fed into the network at time *t*. $W_{r}$, $W_{z}$ and $W_{h}$ are trainable weights of feed-forward connections, whereas $U_{r}$, $U_{z}$ and $U_{h}$ are weights of the recurrent connections. $B_{r}$, $B_{z}$ and $B_{h}$ are bias vectors. $Y_{t}$ indicates the resultant of the GRU model at time *t*, which gives the detected result using the SoftMax activation function, and $W_{o}$, $B_{o}$ represents the weight and bias of $H_{t}$. The error at each time step is calculated using the predicted output $\hat{Y_{t}}$ at each time step and the actual output $Y_{t}$ at each time step and it is given by:

8 $$\begin{aligned} E_{t}=\, & {} -Y_{t}log\left( \hat{Y_{t}}\right) \end{aligned}$$

9 $$\begin{aligned} E=\, & {} \sum _{t}E_{t} \implies E = \sum _{t}-{Y_{t}log\left( \hat{Y_{t}} \right) } \end{aligned}$$

And the total error is calculated by summing up the errors at all time steps, represented in Eq. (9). From the above formulas, the GRU model accomplishes long-distance preservation of valuable particulars by reducing the number of gating units, continuously disposing of unwanted particulars, and using the hidden state to store information dependencies. Although GRU maintains a long-term information dependency, it has a slow convergence rate and low learning efficiency. Therefore, we proposed an optimized GRU that uses a reset gate to optimize the learning structure of GRU and enhance the learning and prediction accuracy.

### MediaPipe

**Figure 2:**
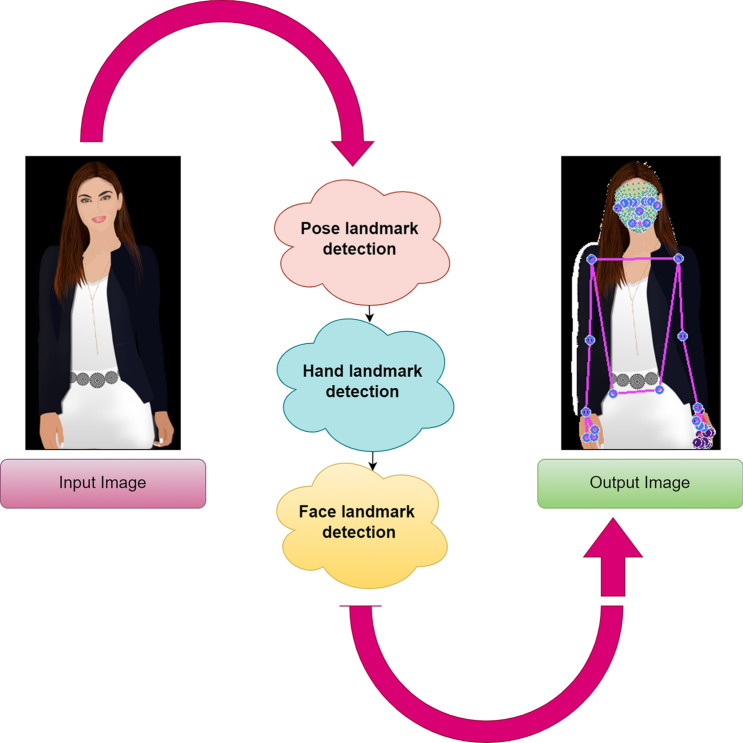
Overview of MediaPipe holistic.

MediaPipe is an open-source framework with a hybrid platform that creates pipelines for processing perceptual data, such as images, videos, and audio. It is an extensive approach employed with ML for hand tracking and gesture recognition in real-time. It provides more hand and finger tracking solutions by accurately detecting the sign gestures. Specifically, we employed a MediaPipe Holistic pipeline to obtain the landmarks from the face, hands, and body pose. Figure 2 clearly outlines the overall functionality.

#### MediaPipe holistic pose landmarks

The MediaPipe Holistic body pose model infers approximately 33 3D landmarks consisting of *x*, *y*, and *z* coordinates on the body from the input image or video using its BlazePose detector and locates the person/pose regions of interest (ROI) within the frame. Using the ROI-cropped frame as input, the pose landmark and division masks within the ROI detect poses successively. Thus, it accurately localizes more key points and suitably fits SLR.

#### MediaPipe holistic hand landmarks

MediaPipe Holistic hands infer approximately 21 3D hand landmarks consisting of *x*, *y*, and *z* coordinates in just a single frame and produce the desired output by combining two models: the palm detection model and the hand keypoint localization model. Initially, the model was employed with a single-shot detector called Blaze Palm. This detector supports the MediaPipe to reduce the time complexity of palm detection given a large dataset of hand sizes in the input image. This model works on the entire image and returns a focused bounding box that highlights the rigid parts, such as palm and fist, for palm detection rather than concentrating on unnecessary objects. Then, the model uses the palm detection output to perform hand keypoint localization. This produced three possible outputs as follows:

- 21 hand knuckle points in a 2D or 3D space.
- Hand flag showing the probability of hand presence in the input image.
- Binary classification of left and right hand.

#### MediaPipe holistic face landmarks

The MediaPipe face mesh is a face geometry solution that calculates 468 3D face landmarks in real-time with a single input camera and not a depth sensor. It works based on two deep neural network models, a detector that computes and operates face locations on a full image and a 3D face landmark model that operates on the computed locations that predict approximate surface geometry using regression. With accurate cropping of the face, data augmentation processes, such as rotation, scaling, and translation are reduced, allowing the network to focus more on coordinate prediction accuracy.

## Proposed methodology

**Figure 3:**
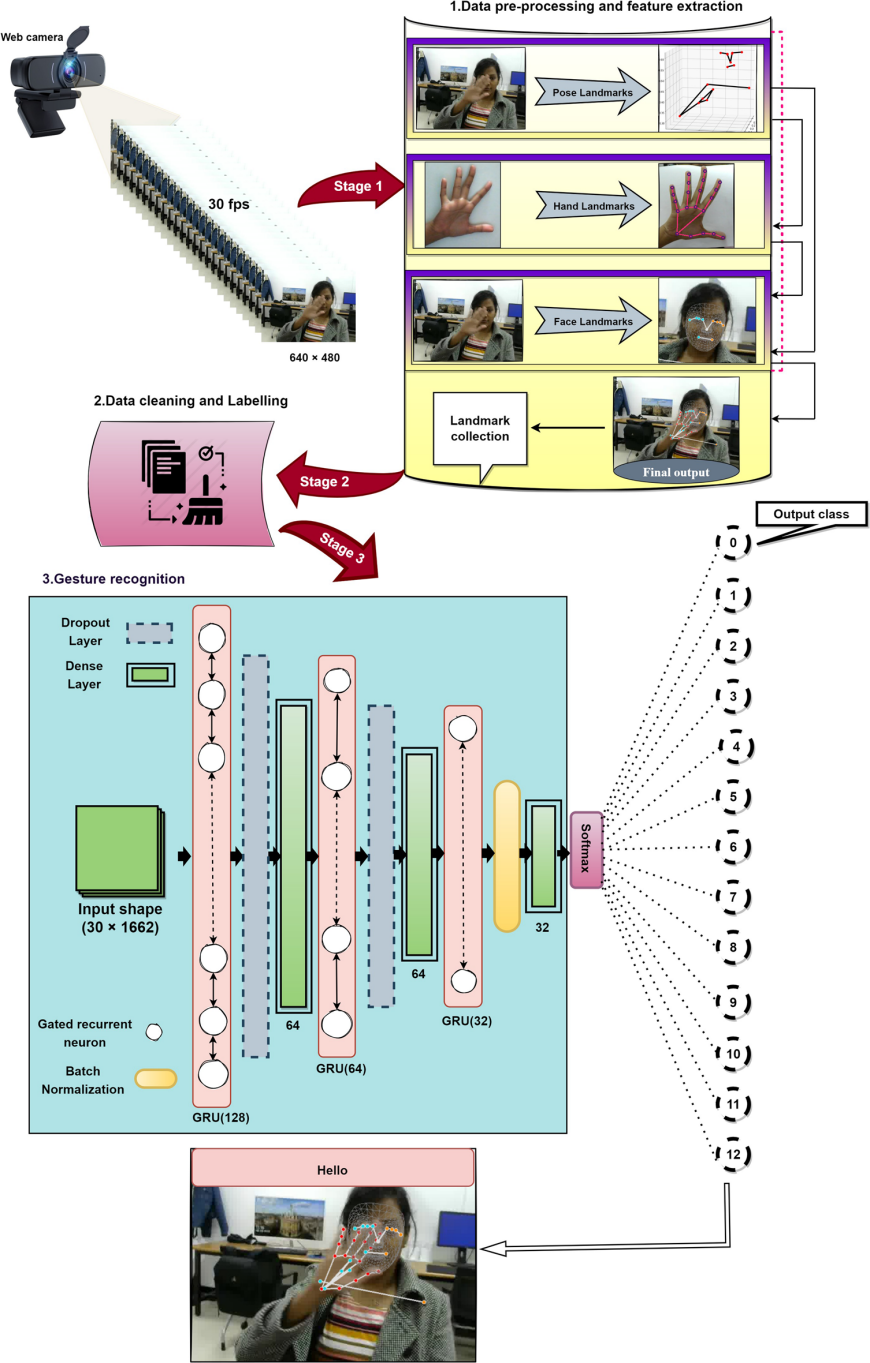
General overview of our proposed architecture.

To accurately recognize the sign gestures and translate them into text, our proposed method comprises three stages: data preprocessing and feature extraction, data cleaning and labelling and gesture recognition. Data preprocessing and feature extraction are carried over by the MediaPipe framework. Here, features from the face, hands, and body are extracted as keypoints and landmarks using built-in data augmentation techniques from sequence of input frames taken from a web camera. In stage 2, the extracted keypoints from stage 1 are saved in a file to identify and remove the null entries from the data, after which data labelling follows. In stage 3, the cleaned and labelled gestures are trained and classified by our MOPGRU model for ISL recognition with the translated sign gestures in the form of text on the screen. Figure 3 shows a general overview of the proposed architecture for an SLR system. For further understanding, the three stages of the proposed methodology are elaborately discussed below.

**Figure 4:**
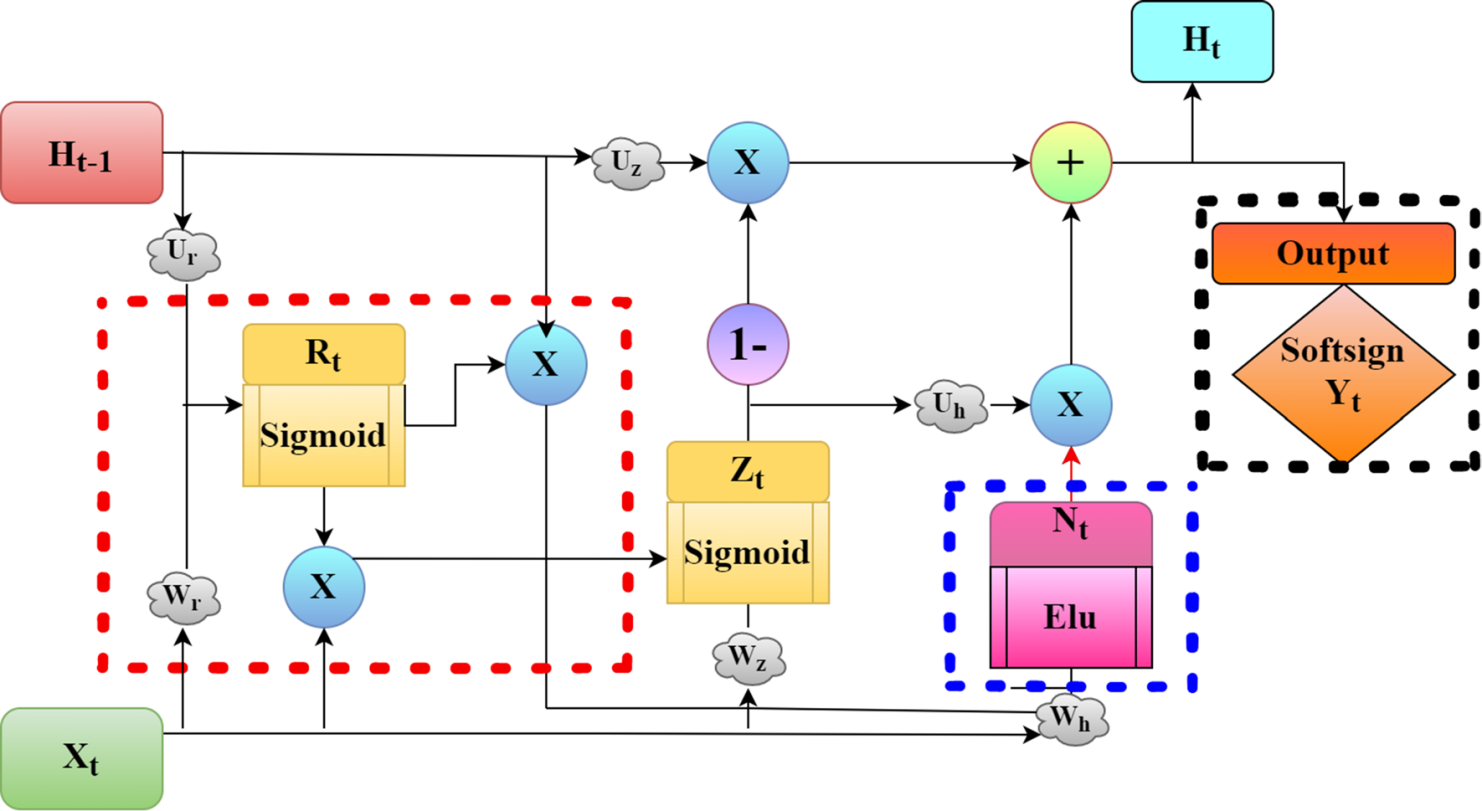
Structure of our proposed GRU cell.

### Stage 1: data preprocessing and feature extraction

For data preprocessing and feature extraction from the image, we applied a multistage pipeline from MediaPipe, called MediaPipe Holistic. For each input frame from the web camera, the MediaPipe Holistic handled individual models for the hands, face, and pose components using a region-appropriate image resolution. The workflow of stage 1 is briefly discussed below:

- The human pose and subsequent landmark model were estimated using BlazePose’s pose detector. Then, three ROI crops for the face and hands (2$\times$) were derived from the inferred pose landmarks, and a recrop was employed to improve the ROI.
- Next, the corresponding landmarks were estimated. To achieve this, the full-resolution input coordinates were cropped to the ROIs for task-specific hand and face models.
- Finally, all landmarks were combined to yield the full 540+ landmarks.

### Stage 2: data cleaning and labelling

After stage 1, the extracted features, that is, the landmark points ($21*3+21*3+33*4+468*3=1662$) per frame are flattened, concatenated and stored in a file to check and remove any null entries from the data. Data cleaning is important since it prevents failed detection of features^56–58^, which occurs when a blurred image is sent to the detector and leads to a null entry into the dataset. Thus, when training occurs with this noisy data, the prediction accuracy is reduced and bias may occur. To fit the obtained data for the next stage of training, testing and validation, labels are created for each class and their corresponding frame sequences are stored.

### Stage 3: gesture recognition

#### Our proposed MOPGRU model

The cleaned and labelled data from stage 2, are then passed to stage 3. The major alteration performed in the standard GRU cell is that its update gate is improved, the candidate memory activation function (tanh) in Eq. (3) is substituted by the ELU activation function, and SoftMax activation function in Eq. (5) by Softsign respectively.

#### Improving the update gate of the standard GRU cell

Considering the problems, such as low learning efficiency, high computational cost, slow convergence rate and incapable of dropping out the unwanted information in one screening with the complex state of time series data of the standard GRU model, we propose a MOPGRU neural network model by improving the update gate; that is, modifying the original update gate input $X_{t}$ to $X_{t}$ multiplied by $R_{t}$. Thus, the update gate is adjusted by obtaining feedback from the output of the reset gate. The opposite effects caused by unnecessary information are profoundly avoided by refining the present input information $X_{t}$ by the reset gate attains faster convergence and efficient in learning. Figure 4 shows the neuronal structure of our proposed GRU cell. The red dashed box represents the changes made in the update gate with the reset gate from the standard GRU; the blue dashed box shows the standard hyperbolic tangent activation replaced with ELU activation and the black dashed box shows the SoftMax activation replaced with Softsign activation.

The formula remains unchanged as mentioned in the standard GRU except for Eqs. (2), (3) and (5). The modified formula for the proposed MOPGRU model is as follows:

10 $$\begin{aligned} Z_{t}=\delta ((W_{z}\cdot X_{t}+U_{z}\cdot H_{t-1})*R_{t}+B_{z}) \end{aligned}$$

Here, the symbols $Z_{t}$ and $R_{t}$ in Eq. (10) hold the same meaning as in the standard GRU cell unit, except in $Z_{t}$ unit structure. The reset gate, $R_{t}$, is multiplied by the input vector $X_{t}$ and then, by the previous time step $H_{t-1}$ in Eq. (10) helped to conceal the state weight, so that the resultant of $R_{t}$ re-screens the present input $X_{t}$ by adjusting $Z_{t}$ to optimize the neuron structure. Our improved GRU cell will not cause any change in computing the derivative of the loss functions as the weights are not changed. Thus, the proposed MOPGRU model makes more sense than the standard GRU, reduces the hidden state and conceals the impoverished gradient to a limited extent. Therefore, our proposed model preserves the information dependency of a longer distance, while producing higher learning efficiency and prediction accuracy.

#### Incorporating ELU

The other change implemented in the standard GRU cell was replacing the candidate memory tanh activation function with the ELU function, as shown in Fig. 4. This is highlighted with a blue dashed box. Thus, Eq. (3) in the standard GRU cell for calculating the candidate memory $N_{t}$ changes to the following form:

11 $$\begin{aligned} N_{t}=ELU(W_{h}\cdot X_{t}+U_{h}(H_{t-1}\odot R_{t}))+B_{h} \end{aligned}$$

The use of the *tanh* activation function in training feed-forward connections, especially in standard GRU, is ineffective as shown in the performance decline when the network has deeper connections^59^. Additionally, it is computationally expensive and has a vanishing gradient due to its exponential operation. Similarly, the rectified linear unit (ReLU) activation has the dying ReLU problem as its derivative is 0 for negative inputs, meaning that weights are not updated during backpropagation, which leads to zero gradients and dead neurons. Additionally, using it for long-range sequences leads to numerical instability because of its unbounded nature^60^.

The ELU activation function given below in Eq. (12) was employed to determine the candidate memory state for the following reasons:

- Using ELU in deep neural networks results in higher classification accuracy and speedy learning^61^.
- It does not suffer from vanishing and exploding gradient problems because of its non-saturating characteristics.
- Since ELU is continuous and differentiable, the problem of dying neurons is solved.
- Compared to other activation functions, ELU achieves higher accuracy and faster convergence in less training time and computational complexity since the negative ELU value permits the mean unit to shift toward 0^61^.

Mathematically, the ELU is defined as,

12 $$\begin{aligned} ELU(x)=\left\{ \begin{matrix} x ,&{} \quad if \; x>0\\ \alpha (exp(x)-1) ,&{}\quad if \; x\leqslant 0 \end{matrix} \right\} \end{aligned}$$

and its corresponding derivative is defined as,

13 $$\begin{aligned} ELU'(x)=\left\{ \begin{matrix} 1 ,&{}\quad if \; x>0\\ ELU(x)+\alpha ,&{}\quad if \; x\leqslant 0 \end{matrix} \right\} \end{aligned}$$

where $\alpha$ is the ELU hyperparameter that controls the value of negative inputs. From Eq. (13), for all positive input values *x*, the function simply returns the corresponding output *Y*. However, if the input *x* is negative, the corresponding output *Y* will be $exp(x)-1$. The output of the derivative function moves closer to one. In order to understand the usage of ELU better, let us consider the gradient equation in the BPTT algorithm^62^:

14 $$\begin{aligned} \frac{\partial E}{\partial W}&= \sum \limits _{k<=t}\frac{\partial E_{t}}{\partial W} \end{aligned}$$

15 $$\begin{aligned}&= \sum \limits _{k<=t} \frac{\partial E_{t}}{\partial \hat{Y_{t}}} \frac{\partial \hat{Y_{t}}}{\partial H_{t}} \frac{\partial H_{t}}{\partial H_{k}} \frac{\partial ^{+} H_{k}}{\partial W} \end{aligned}$$

where

16 $$\begin{aligned} \frac{\partial H_{t}}{\partial H_{k}} = \frac{\partial H_{t}}{\partial H_{t-1}} \frac{\partial H_{t-1}}{\partial H_{t-2}}\cdots \frac{\partial H_{k+1}}{\partial H_{k}} \end{aligned}$$

Thus the total gradient is calculated using the Eqs. (14), (15) and (16) with the weight matrix *W* contains distinctive weights for current input and previous hidden state for each gate. Each Jacobian $\frac{\partial H_{k+1}}{\partial H_{k}}$ is a product of two matrices is a item of two frameworks: the repetitive weight matrix and diagonal matrix composed of the subordinate of non-linearity,ELU, related with the hidden units.In nonappearance of any input, i.e.,$X_{t}=0$, and with the choice of starting conditions, the two-norm of each Jacobian in Eq. (14) is indistinguishably one and the error gradients don’t develop or decay exponential over time.

#### Softsign function for output prediction of the GRU cell

The output layers of the neural network use the SoftMax or Sigmoid activation functions for multivariate or binary classification problems, respectively. Softmax activation is specifically used to normalize the outputs and convert the weighted sum values to probabilities that sum to one by exponentiating the features and scale with the sum of the exponents. Because of its exponential nature, it is computationally expensive and time-consuming to train the model. Hence, we incorporated the Softsign activation function as mentioned in Eq. (17) in the output layer of each GRU neurons as shown in Eq. (18), to reduce the time as it finds the quadratic polynomials rather than exponentially. Additionally, as it is zero-centered, the networks learn effectively and the saturation in the network does not occur easily. Like tanh activation , the Softsign ranges from $-1$ to 1, and is defined as

17 $$\begin{aligned} Softsign (x)=\frac{x}{|x |+ 1} \end{aligned}$$

where |x| is the absolute value of the input point of *x*. Thus, Eq. (5) in the standard GRU cell for output prediction $Y_{t}$ changes to the following form:

18 $$\begin{aligned} Y_{t}=Softsign((W_{o}*H_{t})+B_{o}). \end{aligned}$$

With changes made in Eqs. (10), (12) and (18) the MOPGRU model finally displays the recognized gesture result on the screen with its corresponding English text translation, as shown in Fig. 3 from the output of stage 3.

### Consent to participate

Informed consent was obtained from the subject for publication of identifying information/images in an online open-access publication.

## Experimental results and discussion

### Dataset

A real-time dataset of 30 videos for each sign gesture with 30 frames was created, each with a size of 640 $\times$ 480, using a web camera in various directions and different lighting conditions. There are 13 sign gestures in the dataset, as described in Table 1. We separated the collected dataset in the ratio of 70:15:15 to form the corresponding training, testing, and validation datasets. Thus, from the 900 images for each sign gesture, 780 images were used for the training set and the remaining 120 images were divided equally, providing 60 images each for testing and validation purposes. Additionally, we evaluated our model on two benchmark datasets: the Word Level American Sign Language (WLASL) dataset^63^ and the LSA64 dataset^41^. The WLASL was created for teaching of sign language and hence the data was collected from multiple public resources that includes variety of signing styles and different video backgrounds.The LSA64 dataset contains 3200 videos of 64 isolated sign gestures from the Argentinian sign language which includes verbs and nouns, performed by 10 different people for each word.

**Table 1:** 13 sign gestures and its label.

Labels	Sign gestures (words)
0	Fail
1	Friend
2	Good
3	Hello
4	I love you
5	Like
6	Location
7	Meet
8	Phone call
9	Take care
10	Thank you
11	Think
12	You

### Experimental setting

The simulation was conducted using Python 3.7 version on a desktop computer with 32 GB RAM and an Intel Core i7 processor with a frequency of 3.60 GHz, running on Windows 10 Pro with a 64-bit operating system. The input image was captured using a web camera with a resolution of 720pixels/30 fps of RGB images. A sequential model of nine layers were created. Of the nine layers, three are GRU layers, a batch normalization layer, two dropout layers, and three dense layers. The first layer of GRU accepts a sequence of landmark keypoints (1662) extracted from the frames of each video with the time step of 30 as each video contains 30 frame sequences. For the hidden units at the first time-step, the input-hidden vectors and all elements of the initial hidden state were set to 0.The number of hidden units per layer for the model was set to 128, 64 and 32 respectively. The total number of parameters was approximately 450,445.The dropout ratio was fixed at 20$\%$ for the hidden and fully connected layers. The training data were set to 100 epochs, with 13 gestures in each batch. To optimize the network, the Adam optimization^64^ method was used and was set to $10^{-4}$ with exponential decay rates of 0.9 and 0.999, respectively. The class scores were calculated using the SoftMax activation function with batch normalized input from the fully connected layer. The number of hidden units per layer for the model was set to 128, 64 and 32 respectively. The total number of parameters was approximately 450,445.

### Evaluation metrics

To evaluate the performance of our MOPGRU model, we used the mean squared error (MSE), mean absolute error (MAE), and R-squared or coefficient of determination metrics, as listed in Table 2. MAE denotes the average of the absolute difference between the predicted and actual values in the dataset. It is given by the following formula:

19 $$\begin{aligned} MAE=\frac{1}{N}\sum _{i=1}^{N} |y_{i}-{\hat{y}} |\end{aligned}$$

**Table 2:** Comparison on MAE, MSE, and $R^{2}$ for different models.

Network model	MAE	MSE	$R^{2}$
Simple RNN	4.10	28.90	− 1.38
LSTM	0.75	4.95	0.59
Standard GRU	0.44	1.38	0.83
BiGRU	0.40	2.50	0.79
BiLSTM	0.85	5.35	0.56
MOPGRU	0.22	1.34	0.88

MSE is the average of the squared difference between the predicted and actual values in the dataset and is calculated by the following formula:

20 $$\begin{aligned} MSE=\frac{1}{N}\sum _{i=1}^{N} (y_{i}-{\hat{y}} )^{2} \end{aligned}$$

$R^{2}$ score indicates how well the model fits the given dataset. It indicates how close the predicted value is to the actual data values. The value lies between 0.0 and 1.0, where 0.0 indicates the worst fit and 1.0 indicates the perfect fit. It was calculated using the following formula:

21 $$\begin{aligned} R^{2}=1-\frac{\sum (y_{i}-{\hat{y}} )^{2}}{\sum (y_{i}- \bar{y} )^{2}} \end{aligned}$$

where $\hat{y}$ represents the predicted value of *y*, and $\bar{y}$ denotes the mean value of *y*. Error calculations were performed from Eqs. (19), (20), and (21). From Table 2, we see that the MAE and MSE values are higher for simple RNN, LSTM, Standard GRU, bidirectional GRU (BiGRU), and deep bidirectional LSTM (BiLSTM), which means that the average residual and variance of the residual are high. Previously BiLSTM showed good performance in action recognition^65^, whereas training LSTM and BiLSTM models were not beneficial for our datasets because we had limited data for sequence prediction. Furthermore, we attempted to produce comparatively low prediction errors using our proposed MOPGRU model.

### Quantitative analysis

The classification metrics were used to analyze the quality of prediction for each sign gesture, as shown in Tables 3, 4, and 5, respectively. These qualities include precision, recall, and F1-score^66–68^, which are calculated using four values: true positives (TP), false positives (FP), true negatives (TN), and false negatives (FN). We conducted experiments using different models as mentioned in Table 2 by inserting each model in the place of our proposed MOPGRU layers in the architecture presented in the paper. The number of correctly predicted data points is called accuracy, and ideally, it must be close to one. The accuracy of the SLR system is calculated as follows:

22 $$\begin{aligned} Accuracy = \frac{TP+TN}{TP+TN+FP+FN} \end{aligned}$$

Precision is the number of positive predictions divided by the total number of positive class values predicted, which is called the positive predicted value. High cost of false positive makes precision an important measure to determine. Like accuracy, this must also be close to one. The precision value is calculated as follows:

23 $$\begin{aligned} Precision = \frac{TP}{TP+FP} \end{aligned}$$

A recall is the fraction of positive events predicted correctly by the model and high cost of false negative makes recall an important measure to determine. It is calculated as follows:

24 $$\begin{aligned} Recall = \frac{TP}{TP+FN} \end{aligned}$$

**Table 3:** Precision.

Class label	Simple RNN	LSTM	BiLSTM	Standard GRU	BiGRU	MOPGRU*
0	1	1	1	1	0.5	**1**
1	–	–	–	1	0	**1**
2	1	1	1	1	1	**1**
3	1	1	1	1	1	**0.75**
4	1	0	0	0	1	**1**
5	0	0	0	1	1	**1**
6	0.67	0.67	0.67	1	0	**1**
7	1	0.75	0.75	–	–	**1**
8	1	1	1	1	1	**1**
9	0.5	1	1	1	1	**1**
10	1	1	1	–	–	**1**
11	0	0	0	0	1	**1**
12	1	1	1	0.5	1	**0.92**

Significant values are in [bold].

**Table 4:** Recall.

Class label	Simple RNN	LSTM	BiLSTM	Standard GRU	BiGRU	MOPGRU*
0	0.33	1	1	1	1	**1**
1	–	–	–	0.5	0	**1**
2	1	1	1	1	1	**1**
3	0.5	1	1	1	1	**1**
4	1	0	0	0	1	**0.86**
5	0	0	0	1	1	**1**
6	1	1	1	1	0	**1**
7	1	1	1	–	–	**1**
8	1	1	1	1	1	**1**
9	1	1	1	1	1	**1**
10	1	1	1	–	–	**0.7**
11	0	0	0	0	0.5	**1**
12	1	0.5	0.5	1	1	**1**

Significant values are in [bold].

**Table 5:** F1-Score.

Class label	Simple RNN	LSTM	BiLSTM	Standard GRU	BiGRU	MOPGRU*
0	0.5	1	1	1	0.67	**1**
1	–	–	–	0.67	0	**1**
2	1	1	1	1	1	**1**
3	0.67	1	1	1	1	**0.86**
4	1	0	0	0	1	**0.92**
5	0	0	0	1	1	**1**
6	0.8	0.8	0.8	1	0	**1**
7	1	0.86	0.86	–	–	**1**
8	1	1	1	1	1	**1**
9	0.67	1	1	1	1	**1**
10	1	1	1	–	–	**0.82**
11	0	0	0	0	0.67	**1**
12	1	0.67	0.67	0.67	1	**0.96**

Significant values are in [bold].

The F1-score or the F-measure is the harmonic mean of precision, and recall means it conveys a balance between recall and precision. When there is perfect precision and recall, the F1 score reaches its best value at 1 and can be calculated using the following formula:

25 $$\begin{aligned} F1 = \frac{2TP}{2TP+FP+FN} \end{aligned}$$

Based on the calculations given in Eqs. (22), (23), (24) and (25), we obtain a classification report as shown in Tables 3, 4 and 5. From the classification metrics, the precision, recall, and F1-score of MOPGRU model are almost 1 except for two values in both precision and recall, and four values in F1-score, which are still ideally close to 1. This shows that our model effectively learned data on complete training. In Contrary, other models such as LSTM, Simple RNN, Standard GRU, BiLSTM and BiGRU resulted in good accuracy, but their learning efficiency is not good. As a result, these models did not perform well as they contain many zero metric scores for precision, recall, and F1-score.

**Figure 5:**
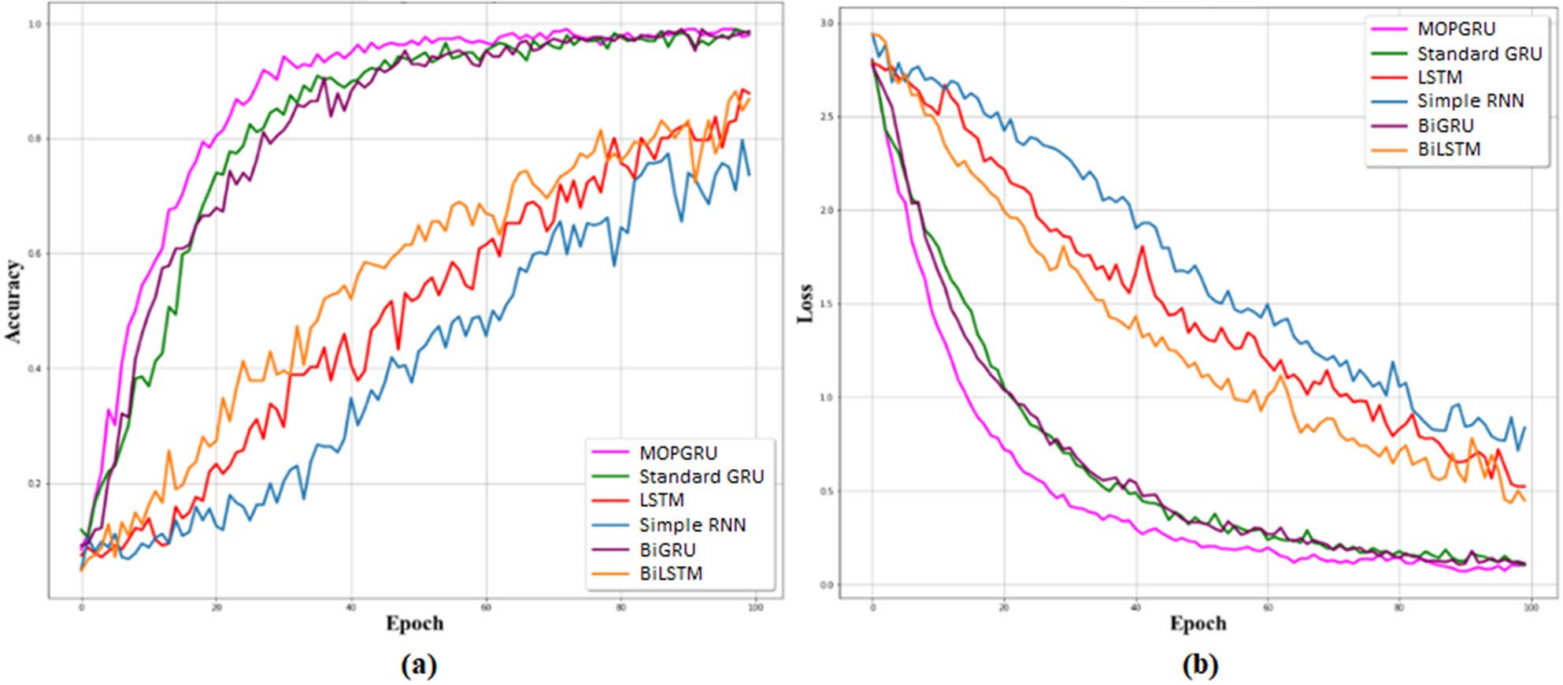
Learning graph with training accuracy and loss for 100 epochs of different models used.

**Figure 6:**
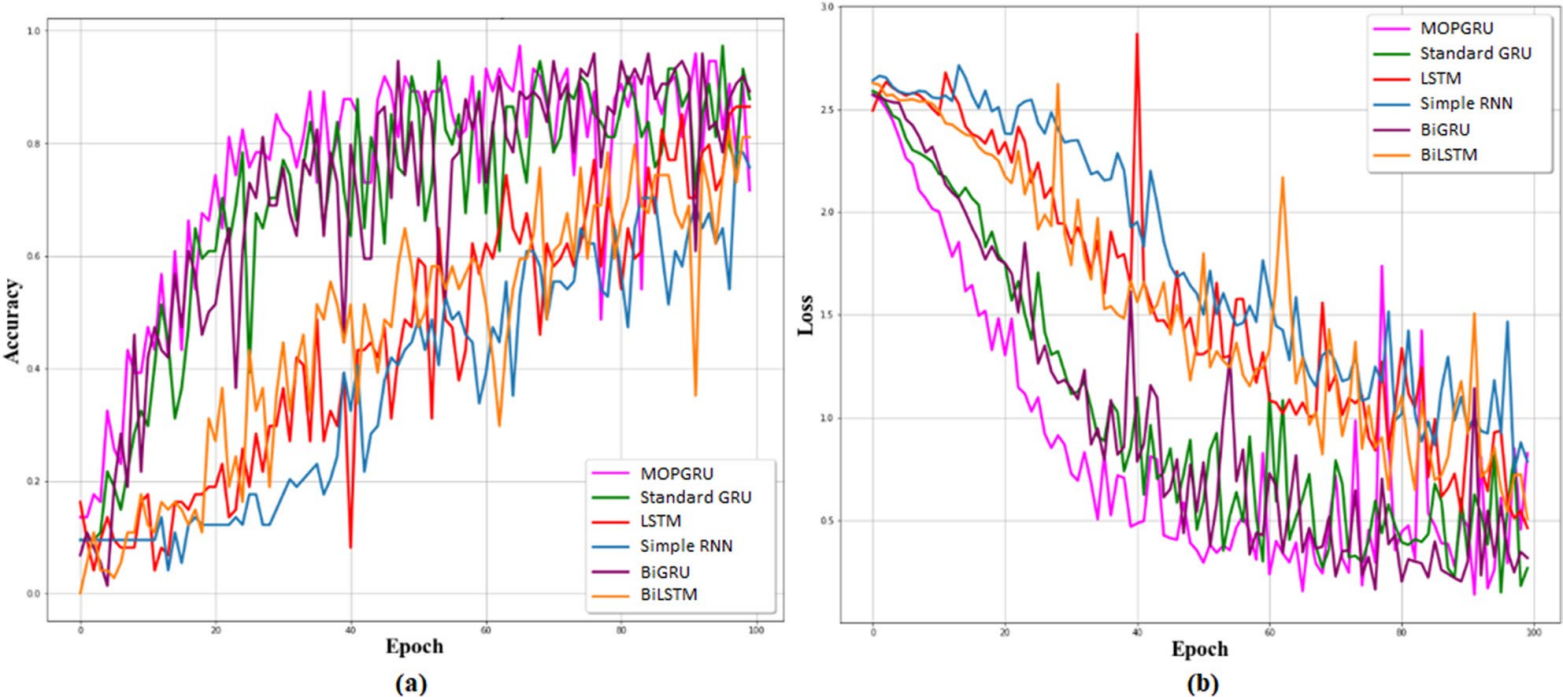
Learning graph with validation accuracy and loss for 100 epochs of different models used.

**Figure 7:**
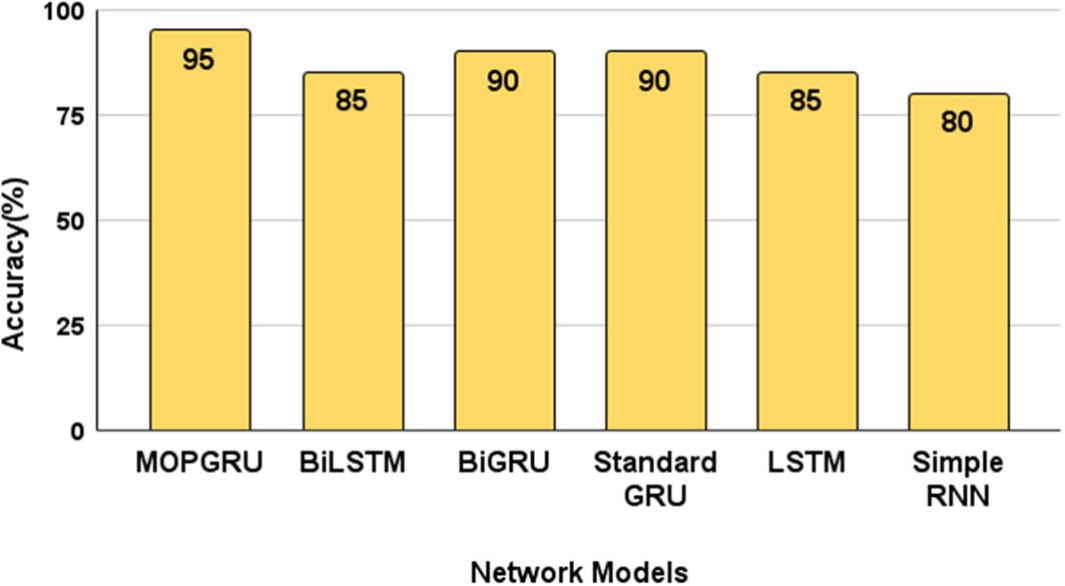
Model comparison in terms of test accuracy in percent ($\%$).

**Figure 8:**
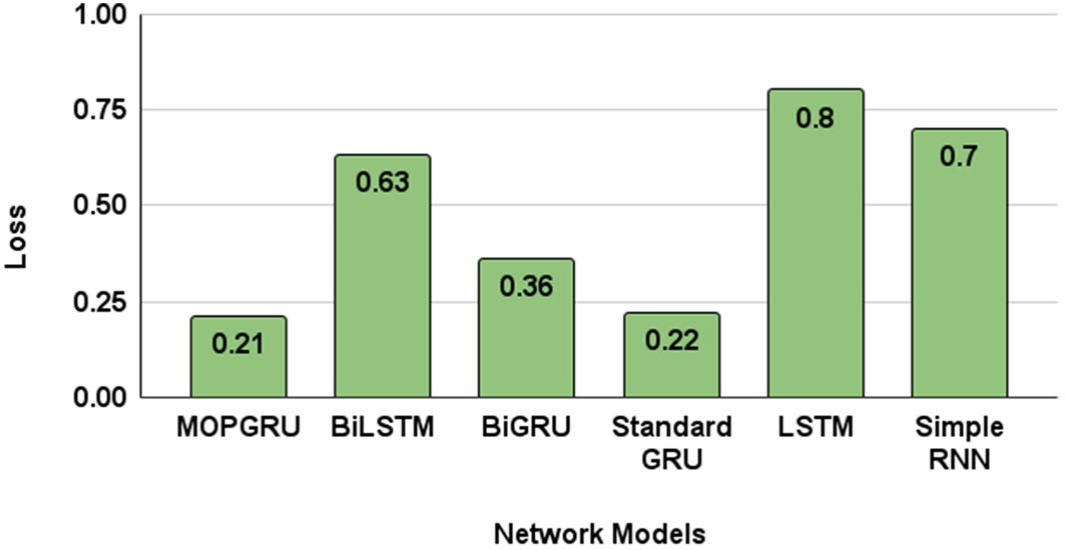
Model comparison in terms of test loss.

Considering the $R^{2}$ values, our proposed MOPGRU outperforms the other three models with the highest score of 0.88, which is closer to 1, indicating that the model has a good fit. However, a negative score in the Simple RNN indicates that the model has the worst fit. Figure 5a and b show the training accuracy and loss of different models and Fig. 6a and b show the validation accuracy and loss of different models used to classify 13 individual sign gestures from the dataset. From Fig. 5, we can conclude that the training is unstable due to the fluctuation between the accuracy and epochs, as well as with loss and epochs. However, in the case of MOPGRU, the training is smooth and faster with efficient data learning. Therefore, our proposed model produces higher performance with minimum loss compared with other models, such as RNN, LSTM, standard GRU, BiGRU, and BiLSTM, respectively. By comparing the models with respect to both the test accuracy and loss, as shown in Figs. 7 and 8, we conclude that our proposed MOPGRU model achieved the highest test accuracy of 95$\%$ compared to other models such as LSTM with 85$\%$, Simple RNN with 80$\%$ standard GRU with 85$\%$ accuracy, BiGRU with 90$\%$, and BiLSTM with 85$\%$ and minimum loss of 0.21 compared to others.

### Comparative analysis

To access the efficiency and performance of our proposed MOPGRU model, we conducted experiments on the two different benchmark datasets LSA64 and WLASL100 respectively and compared our results with different existing state-of-the art models like I3D, Pose-based temporal graph convolutional network (Pose-TGCN) and Pose-based gated recurrent unit (Pose-GRU). We train the model with the same parameters mentioned in^63^ and achieved high recognition accuracy than the existing method. As compared to other models used in the experiment, the learning efficiency and the convergence speed of our proposed MOPGRU was also high with the use of a smaller number of parameters and the activation function during training. Datasets which are existing have their own properties to deal with the isolated word level sign recognition task. However, they fail to capture the complexities of the task due to inadequate amount of instance and signers. Thus we evaluated our model on two benchmark datasets that have adequate amount of instance and signers. The recognition accuracy on both the datasets are presented in Table 6.

**Table 6:** Recognition accuracy achieved by several models on the LSA64 and WLASL100 dataset.

Model	Dataset	Accuracy (%)
Pose-TGCN	WLASL100	55.43
Pose-GRU	WLASL100	46.51
MOPGRU	WLASL100	**63.18**
I3D	LSA64	98.91
MOPGRU	LSA64	**99.92**

Significant values are in [bold].

## Conclusion

In this study, we proposed a MOPGRU model for ISL recognition. Specifically, we modified the update gate of the standard GRU cell by multiplying its output by the reset gate. With our improved update gate mechanism, the output of the reset gate re-screens the information and removes the unwanted information in the data, thereby giving more attention to the important information. We cost-effectively implemented the model, which resulted in improved learning efficiency, prediction accuracy, and information processing capability of the standard GRU neural network. Furthermore, experimental results showed that compared to simple RNN, LSTM, standard GRU, BiGRU, and BiLSTM prediction models, MAE and MSE values of our proposed GRU neural network model were very low with high R-squared values. Therefore, our proposed MOPGRU captured the full information dependency in time series data with a high prediction accuracy of an average of 95$\%$ and a faster convergence speed. Although our model performs well in terms of accuracy and learning efficiency, this study was conducted with a limited dataset. Thus, for future work, we aim to improve our SLR system by expanding the dataset with more vocabulary to predict continuous sign language sentences.

## Data Availability

The datasets generated during and/or analysed during the current study are available from the corresponding author on reasonable request.
